# Mapping Accuracy of Short Reads from Massively Parallel Sequencing and the Implications for Quantitative Expression Profiling

**DOI:** 10.1371/journal.pone.0006323

**Published:** 2009-07-28

**Authors:** Nicola Palmieri, Christian Schlötterer

**Affiliations:** Institut für Populationsgenetik, Veterinärmedizinische Universität Wien, Vienna, Austria; Texas A&M University, United States of America

## Abstract

**Background:**

Massively parallel sequencing offers an enormous potential for expression profiling, in particular for interspecific comparisons. Currently, different platforms for massively parallel sequencing are available, which differ in read length and sequencing costs. The 454-technology offers the highest read length. The other sequencing technologies are more cost effective, on the expense of shorter reads. Reliable expression profiling by massively parallel sequencing depends crucially on the accuracy to which the reads could be mapped to the corresponding genes.

**Methodology/Principal Findings:**

We performed an in silico analysis to evaluate whether incorrect mapping of the sequence reads results in a biased expression pattern. A comparison of six available mapping software tools indicated a considerable heterogeneity in mapping speed and accuracy. Independently of the software used to map the reads, we found that for compact genomes both short (35 bp, 50 bp) and long sequence reads (100 bp) result in an almost unbiased expression pattern. In contrast, for species with a larger genome containing more gene families and repetitive DNA, shorter reads (35–50 bp) produced a considerable bias in gene expression. In humans, about 10% of the genes had fewer than 50% of the sequence reads correctly mapped. Sequence polymorphism up to 9% had almost no effect on the mapping accuracy of 100 bp reads. For 35 bp reads up to 3% sequence divergence did not affect the mapping accuracy strongly. The effect of indels on the mapping efficiency strongly depends on the mapping software.

**Conclusions/Significance:**

In complex genomes, expression profiling by massively parallel sequencing could introduce a considerable bias due to incorrectly mapped sequence reads if the read length is short. Nevertheless, this bias could be accounted for if the genomic sequence is known. Furthermore, sequence polymorphisms and indels also affect the mapping accuracy and may cause a biased gene expression measurement. The choice of the mapping software is highly critical and the reliability depends on the presence/absence of indels and the divergence between reads and the reference genome. Overall, we found SSAHA2 and CLC to produce the most reliable mapping results.

## Introduction

Technological advances have revolutionized the analysis of the transcriptome, the set of genes expressed in a given tissue. Currently, a broad range of techniques is widely used for expression profiling, but each available technique has its specific limitations.

The first widely used microarrays were based on cDNA sequences [1]. PCR amplified cDNA fragments are spotted at a high density (10–50 spots per mm^2^) onto a microscope slide and probed against a labelled target. This technique offers the advantage that it is rather insensitive to mismatches between the probe and the cDNA sequence. The drawback of these probes is that they are very sensitive to cross-hybridization; hence it is impossible to contrast the expression pattern of genes with a similar sequence (e.g.: members of a multigene family). Microarrays with multiple short oligos per gene are used for most commercial gene expression platforms (e.g. Affymetrix). While longer oligos offer the advantage of more reliable hybridization, they are more prone to cross-hybridization than platforms using shorter ones.

Despite considerable effort to develop oligos with similar hybridization properties [2], the hybridization behaviour is very complex. The same target RNA molecule may hybridize with different efficiency resulting in different hybridization signals [3]. Hence, due to the sequence specific hybridization behaviour, the above-mentioned techniques are not well suited for measuring absolute expression levels and they are mainly used to compare the expression pattern of different samples relative to each other. As only relative gene expression levels can be inferred reliably, studies requiring measures of absolute gene expression are best advised to rely on different methods for expression profiling.

Expressed sequence tag (EST) sequencing is a very powerful, but also expensive method to measure gene expression. Building a cDNA library and sequencing a large number of clones provides a good overview on the absolute expression level, as the frequency of a given transcript is proportional to its expression level. Hence, it is possible to compare the expression of different genes without having to rely on the assumption of homogeneous hybridization behaviour. The high costs of EST sequencing however, make an extension to many species and tissues impractical. An alternative to EST sequencing is the Serial Analysis of Gene Expression (SAGE) technique [4]. Rather than sequencing individual cDNAs, for SAGE short 14 bp cDNA tags are concatenated and jointly sequenced. This allows an in depth analysis of gene expression at significantly lower, albeit still considerable costs.

The greatest challenge for all these methods is the comparison of gene expression across species. Despite that cDNA arrays potentially offer the advantage of only minor effects of mismatches, some studies of closely related species showed that cross-species expression profiling using one common cDNA array is complicated and may yield spurious results [5]. Furthermore, the well-known heterogeneity in evolution rates among genes further complicates the comparison of expression divergence between two species across genes. Recently, a modification of the cDNA arrays has been proposed, which requires a cDNA probe for each gene in every species included in the study [5], [6], which makes the generation of the arrays extremely labour intensive. Oligo arrays have also been used for cross-species expression profiling by restricting the analysis to those probes that were perfectly conserved between the species analyzed [7]. For obvious reasons, this approach is limited to closely related species for which a fully sequenced genome is available. In principle SAGE could be applied to any species, but the identification of the gene corresponding to a given SAGE tag does not only require a fully sequenced genome, but also a reliable gene annotation including a characterized 3′ UTR, as a large proportion of the SAGE tags is located in the 3′UTR [8]. Even the modifications of the traditional SAGE method, which result in longer tags [9] are not sufficient for poorly annotated genomes. Similar to SAGE, massively parallel signature sequencing approaches (MPSS), which result in 16–20 bases [10], suffer from the problem to assign the short sequences unambiguously to incompletely annotated genomes. EST sequencing is probably the most general method, but a broad application is prohibited by the high costs.

The increased read length of the second-generation sequencing technologies could potentially overcome most of the disadvantages mentioned above. Depending on the platform used, the read length of a single sequence is at least 35 bp, which provides a substantial improvement in the ability to accurately identify the gene corresponding to the short sequence read. Hence, by sequencing random pieces of cDNA molecules, massively parallel sequencing potentially provides an enormous potential to quantify gene expression.

Recently, Torres et al. [11] showed that the sequencing of 3′ ends of randomly sheared cDNA molecules provides an excellent tool for quantifying gene expression. Using the 454 sequencing technology they unambiguously mapped 97% of the sequence reads and showed that the obtained expression profiles were highly reproducible. Nevertheless, compared to the competing massively parallel sequencing technologies (Illumina, SOLiD), the cost per base with the 454 sequencing technology is about one order of magnitude higher. This raises the question if the shorter reads obtained with the more affordable sequencing technologies are sufficient for gene expression studies – i.e.: could they be mapped to the corresponding genes with a similar efficiency as longer sequence reads?

In this report we compare the mapping efficiency of reads of different length. We show that for smaller genomes, such as yeast and Drosophila, even the shortest 35 bp reads perform well and do not result in a major bias. For larger genomes, such as humans, a considerable bias could be observed for 35 bp reads.

## Results

We were interested to study how the accuracy of mapping short reads is affected by size of the reads, complexity of the reference genome, and the mapping algorithm used. As quantitative transcript profiling by massively parallel sequencing is potentially affected by the accuracy of the mapping of short reads, we performed an in silico analysis to evaluate this.

We generated in silico reads from *Saccharomyces cerevisiae* (yeast), *Drosophila melanogaster* (fly), *Arabidoposis thaliana*, and *Homo sapiens* (human) transcripts. Some genomic regions are occasionally used to encode for multiple transcripts, by either overlapping or nested genes. As this phenomenon would prevent the unambiguous mapping of reads to one gene, we only used genes that were encoded by a different genomic stretch of DNA.

**Figure 1:**
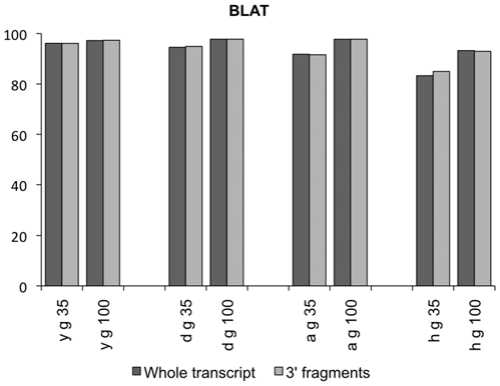
Percentage of correctly mapped reads with BLAT, using reads of 35 bp and 100 bp length, derived from 3′-fragments or whole transcripts. Reads are mapped against the genome of the corresponding species.

Two methods for quantitative expression profiling by massively parallel sequencing have been described: whole transcript sequencing or sequencing of randomly broken 3′ fragments. We focus on the latter method, as it does not require an adjustment of the transcript length. Nevertheless, we obtained qualitatively similar results for both methods (Figure 1).

For every gene we selected the longest transcript, which was randomly sheared. After capturing the 3′ ends, we obtained the short reads from the 5′ end of the captured fragment. We generated sequence reads from four species and mapped them either to the transcriptome or genome using six different programs: BLAT [12], SSAHA2 [13], Bowtie [14], SeqMap [15], MAQ [16] and CLC NGS Cell [www.clcbio.com]. Then we classified the reads in two categories:

- Mapped: reads that mapped to a unique position in the genome (see Material and Methods)
- Correct: reads that mapped to the correct position.

**Figure 2:**
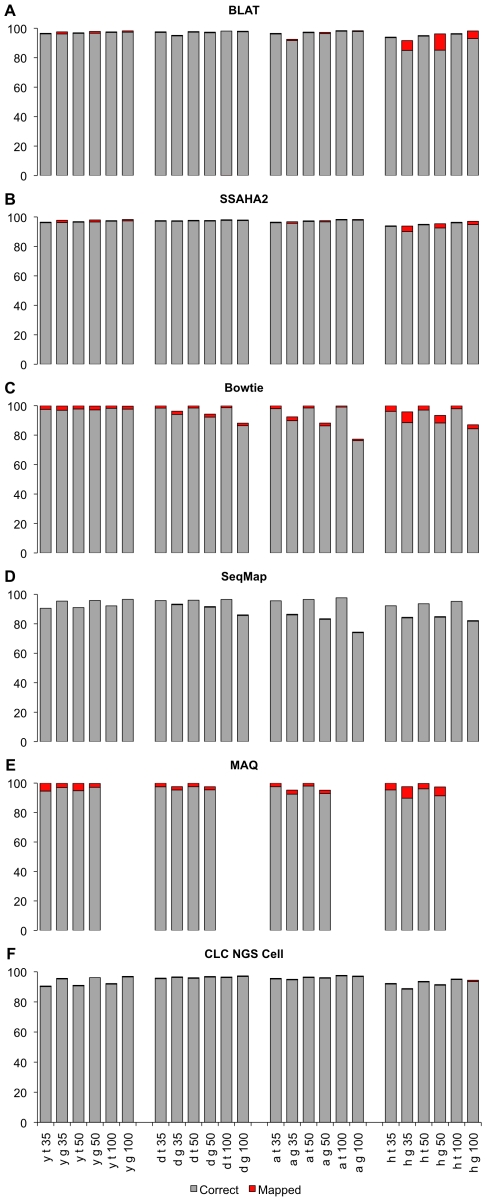
Percentage of mapped and correctly mapped reads with different programs: a) BLAT, b) SSAHA2, c) Bowtie, d) SeqMap, e) MAQ, g) CLC NGS Cell. In each simulation the labels for each bar indicate in the order: organism (y = *S. cerevisiae*, d = *D. melanogaster*, a = *A. thaliana*, h = *H. sapiens*), mapping against the transcriptome (t) or the genome (g), read length (35 bp, 50 bp, 100 bp).

Overall, we found that the mapping accuracy was high. Even the short 35 bp reads were mapped with a high precision. BLAT, SSAHA, SeqMap and CLC mapped almost all reads correctly. Only BLAT had a notable fraction of almost 7% of the 35 bp reads, which were incorrectly mapped against the human genome (Figure 2A). In the case of an ambiguous match Bowtie and MAQ randomly assign reads to one of the targets, which results in a higher proportion of incorrectly mapped reads (Figures 2C, 2E). Hence, neither Bowtie nor MAQ should be used for mapping reads to quantify gene expression.

While for species with a low complexity almost no difference in mapping accuracy could be noted between short 35 bp and long 100 bp reads, for species with a more complex genome the mapping improved with longer reads. In humans and Arabidopsis both the mapping accuracy as well as the number of reads mapped increased with read length. Furthermore, we found that mapping against the transcriptome was more effective and reliable than mapping against the genome.

Nevertheless, we found some notable exceptions to these general trends. First, in yeast MAQ and CLC mapped a larger proportion of the reads to the genome than to the transcriptome and we could not identify the reason for this counter-intuitive result (Figures 2E, 2F). Second, with Bowtie and SeqMap an increase in read length resulted in a decreased mapping efficiency to the genomic reference (Figures 2C, 2D). This observation is the result of a larger proportion of exon-intron junctions included in the longer sequence reads. As Bowtie and SeqMap do not map reads with more than three mismatches between the read and the reference sequence, reads spanning exon-intron boundaries have frequently more than three mismatches and are therefore not mapped. We validated this hypothesis by mapping reads that did not span two exons against the genome of *D. melanogaster* (see Material and Methods) and we observed an increase of mapping accuracy as the read length increases (Table S2).

**Figure 3:**
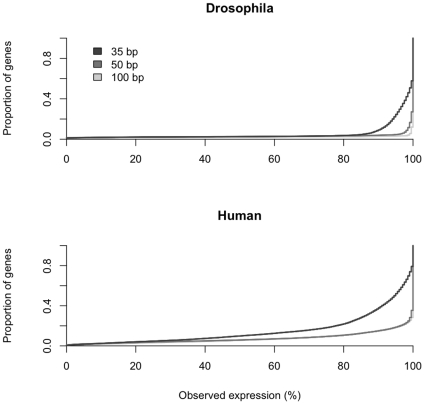
Cumulative distributions of the observed expression with 35 bp, 50 bp and 100 bp reads of *D. melanogaster* and *H. sapiens* mapped against the corresponding genomic sequence.

For expression profiling the incomplete mapping of reads only becomes a problem if genes are differentially affected. If all genes have approximately the same number of reads that cannot be mapped, no bias would be introduced. We evaluated this by using BLAT, as this mapping tool was found to be sensitive to read length and genome complexity and should thus show the most pronounced effect. Figure 3 shows that for the majority of genes the inferred expression level does not deviate from the expectations. Nevertheless, a non-negligible number of genes deviated to a variable degree from the expectations. In *D. melanogaster* 97% of the genes deviate less than 10% from the true expression level for 100 bp reads and 91% for 35 bp reads. For humans only 85% (100 bp reads) and 65% (35 bp reads) of the genes deviate less than 10%. The differences are even more pronounced by considering the genes with more than 50% difference in expression, thus we systematically studied this effect for different species and mapping tools (Table 1). For yeast and *D. melanogaster* no major difference was detected among the read lengths tested. For humans and Arabidopsis, the bad performance of Bowtie and SeqMap for long reads is very prominent, but for CLC, SSAHA2 and BLAT the proportion of genes with more than 50% deviation from the expected expression intensity was substantially decreased (Table 1).

**Table 1:** Percentage of genes showing a reduction in observed gene expression by more than 50% when mapped against a reference genome.

Genome	Read length	BLAT	SSAHA2	Bowtie	SeqMap	MAQ	CLC
**Yeast**	35 bp	3.8%	3.8%	2.5%	4.2%	2.5%	4.2%
	50 bp	3.3%	3.4%	2.2%	3.8%	2.3%	3.8%
	100 bp	2.6%	2.7%	1.8%	3.1%	NA	3.0%
**Drosophila**	35 bp	2.5%	2.5%	2.0%	3.0%	2.0%	3.0%
	50 bp	2.7%	2.4%	2.0%	2.9%	2.0%	2.8%
	100 bp	2.1%	2.1%	5.9%	6.4%	NA	2.6%
**Arabidopsis**	35 bp	3.1%	3.0%	2.0%	4.1%	1.9%	3.6%
	50 bp	2.4%	2.4%	2.6%	5.4%	1.6%	2.9%
	100 bp	1.7%	1.7%	17.1%	19.7%	NA	2.0%
**Human**	35 bp	11.5%	7.6%	6.1%	10.0%	5.8%	9.2%
	50 bp	12.8%	5.9%	5.6%	9.0%	4.7%	7.3%
	100 bp	5.5%	4.5%	13.8%	16.2%	NA	5.5%

Interestingly, irrespective of the read length, we found a small proportion of genes (1.3% in Drosophila) which did not recover a single mapped read. We reasoned, that this observation could be attributed to recent gene duplications, which resulted in almost identical gene copies preventing unambiguous mapping. To test this hypothesis, we aligned the full transcripts of those genes for which we did not map a single read against the genome of the corresponding species. Consistent with the gene duplication hypothesis, we found two equally good hits for all the genes tested. Most importantly, our analysis indicated that even reads longer than 100 bp would not have improved the mapping accuracy for these duplicated genes.

**Figure 4:**
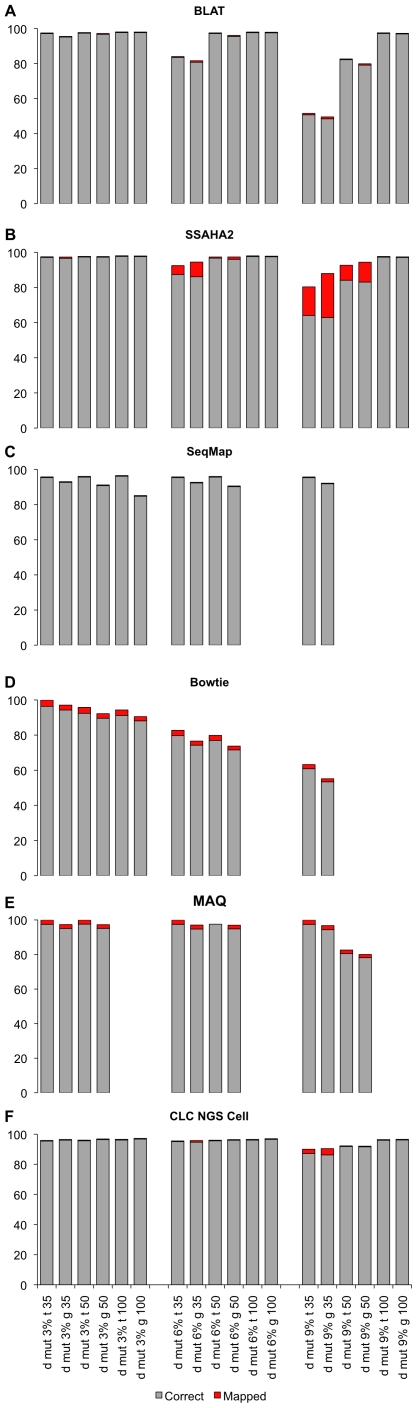
Percentage of mapped and correctly mapped reads with different levels of mutation (3%, 6%, 9% of the total length of each read are mutated).

So far, we only considered sequence reads generated from published genomes, thus no mutations were expected, which could further complicate the mapping of the reads. Nevertheless, many expression analyses focus on individuals with unknown genotypes. Hence, we evaluated the influence of base substitutions and insertions on the mapping accuracy. For simplicity, this analysis was conducted only in *D. melanogaster*. We mutated 3, 6 and 9% of the bases in a read and mapped the mutated reads to the original genome/transcriptome (Figure 4). The mapping of 100 bp reads was almost not affected by mutations. Even with 9% mutations, only a very limited effect on the mapping accuracy was noted. We point out that for 100 bp reads with 9% of mutation the comparison is possible only within the programs BLAT, SSAHA2 and CLC, because the mapping was not feasible with other programs due to high divergence of the reads. For 50 bp reads up to 6% of mutation did not have an effect on the mapping accuracy for all programs, except for Bowtie, which could not deal well with the divergent reads. The most pronounced effects were seen for 35 bp reads. While 3% mutations did not affect the mapping, 6% and 9% mutations had a substantial effect. Interestingly, we noted a marked difference in the performance among the programs used. BLAT could only map about 50% of the reads and SSAHA2 mapped more than 20% of the reads incorrectly. The best results were obtained by MAQ and SeqMap, which still mapped a very high proportion (>90%) of the reads correctly.

**Figure 5:**
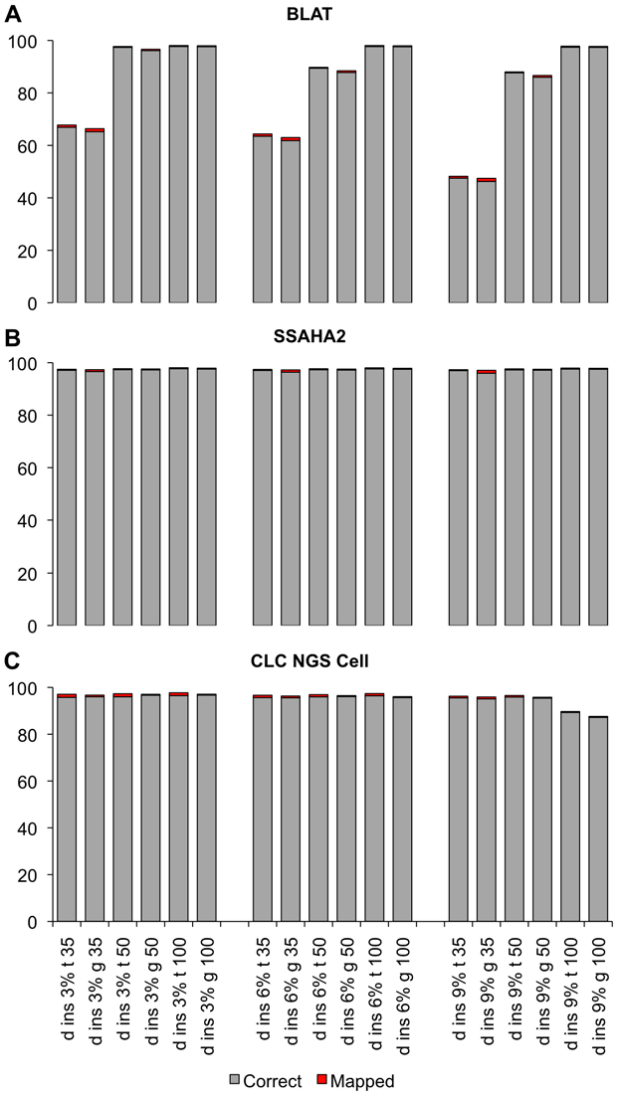
Percentage of mapped and correctly mapped reads with a single insertion of length equal to 3%, 6% or 9% of the total length of each read.

We also tested the effect of insertions by mapping *D. melanogaster* reads containing a single insertion of variable size (3, 6, 9% of the read length) in a random position. This analysis could be only conducted with BLAT, SSAHA2 and CLC (Figure 5), because the other programs cannot perform gapped alignments. While Bowtie also allows insertions, the high demand on CPU time prevented us from a systematic evaluation of this software. Already the insertion of a single base dramatically lowered the mapping efficiency of BLAT (from 97% to 67%). SSAHA2 and CLC were almost not affected by the presence of insertions, irrespective of their length. Nevertheless, SSAHA2 performed slightly better than CLC, which showed a small proportion (9.8% for a 9% insertion in 100 bp reads) of incorrectly mapped reads.

**Figure 6:**
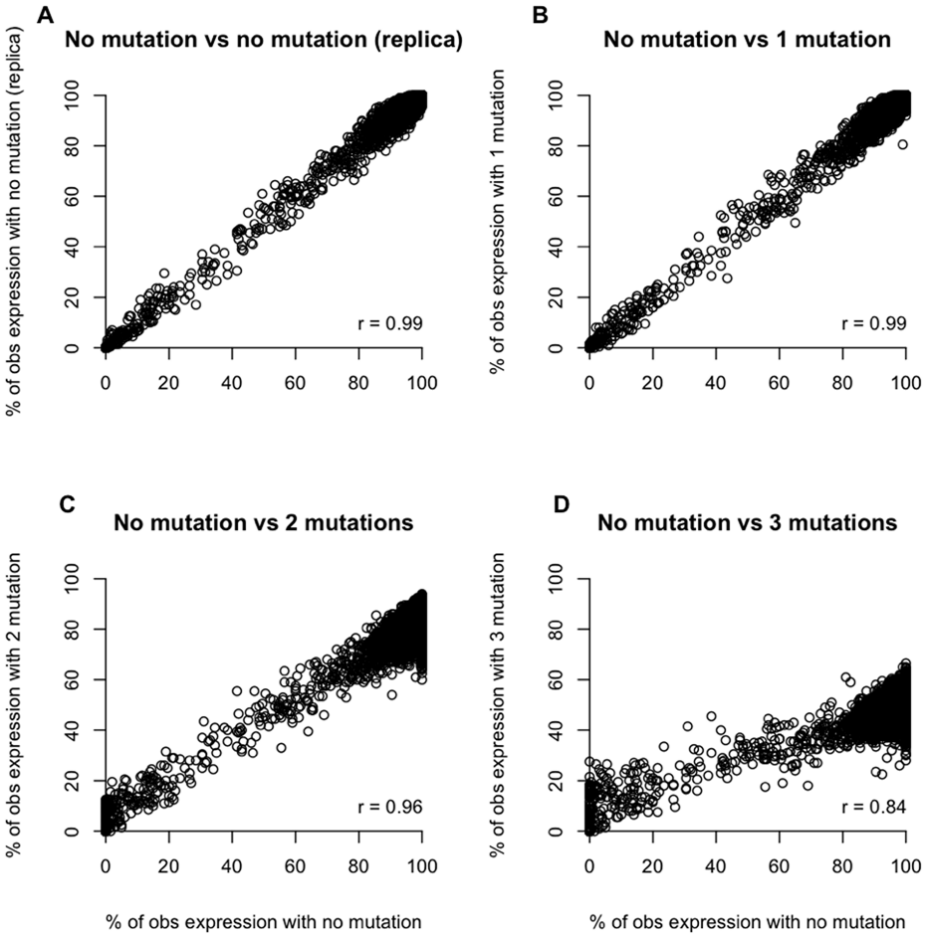
Stochastic variability between observed expression profiles (*D. melanogaster*, short reads mapped with BLAT against the genomic sequence); a circle represents each gene. A) Two independent runs of 35 bp reads without mutations, B) 1 mutation vs. no mutation, C) 2 mutations vs. no mutation, D) 3 mutations vs. no mutation. r indicates the correlation coefficient between the observed expression levels.

We were interested to test if some genes are more prone to suffer from the lower mapping accuracy of mutated reads than others, as this would result in a further bias in gene expression. Panel A in Figure 6 shows the variance in the observed e_obs_ by two independent “sequencing runs”, for which we generated two independent sets of fragments and mapped them to the genome with BLAT, as this mapping algorithm was found to be highly sensitive to mutations. The remaining panels show the comparison of e_obs_ for no mutations to reads with 3%, 6% and 9% mutated bases respectively. Interestingly, e_obs_ for reads with 3% and 6% mutations were highly correlated with e_obs_ from reads without mutations. Only reads with 9% mutations showed a pronounced reduction in the correlation coefficient. Thus, up to 6% sequence divergence no pronounced gene specific effects could be detected, suggesting that we found no evidence for heterogeneity in the effect of mutations on the mapping efficiency.

**Figure 7:**
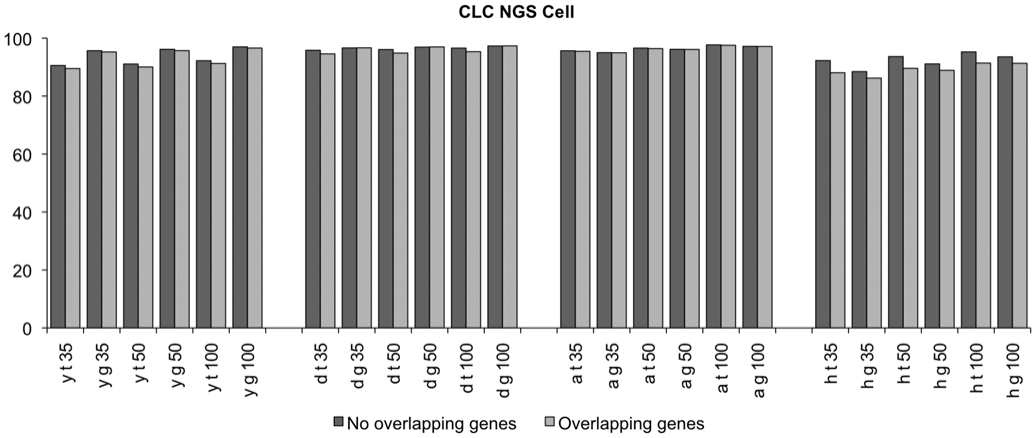
Percentage of correctly mapped reads with CLC NGS Cell. Reads are generated from a transcript database in which the longest isoform is chosen, but overlapping transcripts are not removed.

As our data set only contained non-overlapping genes, we repeated the mapping with CLC, using for each organism a cDNA database in which overlapping genes are not removed, in order to quantify the impact of overlapping genes. As expected we obtained a decrease in mapping efficiency (Figure 7). This is caused by more ambiguities in the mapping, as there are more genes that share partially the sequence. The impact was marginal for Drosophila, Arabidopsis and yeast, but for human there is a decrease of more than 2% in the genome mapping analysis).

Given that expression analyses produce a large number of reads, we evaluated not only the mapping accuracy, but also in the mapping speed of the programs used. For each program we determined the required CPU time on a 2×2.8 GHz Quad-Core Intel Xeon Mac Pro computer for 100,000 reads (100 bp), mapped against the three different species. The fastest mapping software is Bowtie, followed by CLC and SSAHA2. BLAT and SeqMap are the slowest ones (Table 2). Additionally, we report in table S3 a summary of all the relevant features of the mapping tools used.

**Table 2:** Times (in seconds) for mapping 100,000 reads of 100 bp vs genome with different programs in the four organisms analyzed.

Program	Yeast	Drosophila	Arabidopsis
**BLAT**	287	2868	1721
**SSAHA2**	29	107	76
**Bowtie**	50	6	8
**SeqMap**	16	1260	958
**MAQ**	50	470	376
**CLC NGS Cell** *	376	1232	856

* typically the speed is improved by the parallel use of all processors of the computer.

## Discussion

Massively parallel sequencing offers an enormous potential for gene expression profiling. With the number of sequence reads being proportional to the transcript abundance, it is possible to make reliable absolute quantification of transcripts. Hence, the comparison of gene expression between species should be possible by massively parallel sequencing. Currently, researchers are facing the decision between more economic short reads and longer reads. The goal of this study was to compare both approaches with respect to their potential for accurate expression profiling. Apart from technological problems [11], the major challenge is the accurate mapping of the sequence reads to the corresponding gene.

Consistent with previous results [11], we showed that 100 bp reads produce highly accurate mapping results, almost independent of the mapping software used. Even mutations and indels did not show a major effect on the mapping accuracy. If adequate mapping software is used, 50 bp reads produce already highly reliable results for species with a small genome. For humans, however, sequence reads of 35 bp and 50 bp do not yield trustworthy results. Even without mutations, a considerable fraction of the reads were not mapped and more than 10% of the genes differed more than 50% for the true expression level.

The influence of sequence divergence on mapping accuracy is conceptually more difficult to handle. As genes differ in their sequence conservation, this will generate heterogeneity in mapping accuracy among genes that is difficult to predict. Hence, we restricted our analysis to a more general analysis of the impact of mutations and insertions on the mapping accuracy. To do so, we mutagenized each sequence read independently from the others. For 100 bp reads, sequence divergence had almost no effect on the mapping accuracy, even if up to 9% of the bases in the read were mutated. Also a single mutation in 35 bp reads or 3 mutations in 50 bp reads had almost no effect on the mapping accuracy. This suggests that even for polymorphic species, such as *D. melanogaster*, which has about 3% silent site polymorphism [17], short 35 bp reads will be sufficient to cover intraspecies variation without the loss of mapping accuracy. Interspecific comparisons are more problematic when the genome of one of the species is not known. The silent sequence divergence of the two closely related species *D. melanogaster* and *D. simulans* is slightly above 13% [17], suggesting that even the availability of a reference sequence from a close relative does not guarantee a reliable gene expression measurement with short reads. If the right software tool is used, insertions were not found to affect the mapping quality. Nevertheless, clustering of substitutions and indels [18] will probably further reduce the mapping efficiency of a large number of reads.

Based on these results, we conclude that gene expression studies in yeast and Drosophila could be reliably performed with 50 bp reads, even when some sequence divergence between the reference genome and the transcriptome is expected. For humans, and to some extent for Arabidopsis as well, 100 bp reads are preferable.

One important result of our study is the highly heterogeneous performance of the different mapping software tools. While Bowtie was undoubtedly the fastest mapping program, it performed very poorly with mutations and could not deal with insertions. Like Bowtie, SeqMap cannot deal with indels and the limitation to three mismatches strongly affects the mapping of 100 bp reads to genomic DNA. MAQ cannot map reads with indels. One further limitation of Bowtie and MAQ is the random assignment of equally well mapped reads to the corresponding genes. Unless, both genes are expressed at a similar level, this procedure will cause wrong expression estimates and should thus avoided. BLAT is a more general program, which could deal with insertions, but in the presence of mutations the mapping accuracy was only reasonably good for 100 bp reads. CLC and SSAHA2 were found the two programs that produced the most accurate results for most parameters tested. The relatively high rate of incorrectly mapped 35 bp and 50 bp reads with 9% mutations detected for SSAHA2, renders CLC the most versatile and accurate program in our comparison. Nevertheless, we would like to point out that mapping performance of all programs is severely affected by the choice of mapping parameters. As we tested a broad range of species and read lengths, we typically used only the recommended parameter settings. We cannot rule out that some optimization of the mapping parameters would have altered our conclusions about the mapping performance of the software tested.

Irrespective of the software used, we found that a given fraction of genes recovered fewer reads than expected. This under-representation of mapped reads is caused either by gene duplication or repetitive sequences in the transcript. While longer reads reduce this problem to some extent, for genes that were duplicated very recently even reads longer than 100 bp will not improve the situation. We propose a simple correction, which should account for the gene specific recovery of sequence reads. The expected e_obs_ could be determined in a similar procedure as reported here. If the observed counts are weighted by e_obs_ it is possible to account for the bias.

The results of our study are more general than just expression profiling by massively parallel sequencing. Recently, it has been suggested that massively parallel sequencing could also be used to estimate allele frequencies from DNA pools [19]. The accurate genome wide estimate of allele frequencies depends also on a highly accurate mapping of sequence reads. Our results show that the mapping accuracy will be highly dependent on the choice of the software, but also read length and genome complexity will have an effect. While paired end reads, substantially improve the mapping accuracy, our results also suggest that for species with a compact genome, such as *D. melanogaster*, a very high mapping accuracy could be obtained with 50 bp reads.

It is important to keep in mind that all our simulations used tags generated from the sequence to which the reads were mapped. Experimental data, however, may also contain reads for which no reference sequence is known. This could be either due to an incomplete genomic sequence or the presence of other organisms, such as Wolbachia and Viruses. In such cases fewer reads will be mapped and more reads may be incorrectly mapped.

Finally, we would like to point out that our analyses did not account for sequencing errors, as we do not think that they have a severe effect on expression profiling, as the position of the reads is governed by random breakage of the cDNA. Thus, even preferential mutations at the end of a read will not result in a systematic bias. Nevertheless, other biases caused by the sequencing process, such as a GC-content dependent production of reads [20], could also have a substantial effect on the estimated gene expression levels and these are not considered here.

## Materials and Methods

### Data

Sequences of transcripts and chromosomes were obtained for *S. cerevisiae* and *H. sapiens* from the Ensembl database (www.ensembl.org), *A. thaliana* from the Arabidopsis Information Resource (http://www.arabidopsis.org/) and *D. melanogaster* from FlyBase release 5.4 (http://flybase.bio.indiana.edu/).

From the transcripts files we selected the longest isoform for all the protein coding transcripts (mRNA). To avoid the complication of overlapping genes, we removed all the genes that overlap at least with another gene. The number of transcripts before and after filtering is shown in Table S1.

### Generation of sequence reads

We mimicked the generation of sequence reads from cDNA sequences as described in Torres et al. [11]. In brief, Torres et al. [11] randomly sheared the double stranded cDNA molecules, recovered the 3′ fragments and sequenced them from their 5′ end. They observed a pronounced bias against fragments shorter than 80 bp and longer than 300 bp. Thus, we used the experimentally derived distribution of 3′ fragment size distribution to generate 200 sequence reads from each transcript. We generated reads with three size classes, 35, 50 and 100 bp.

We also used a uniform distribution between 38 bp and 1000 bp to measure gene expression and obtained almost identical results (not shown). Hence, the results based on the experimental distribution of 3′ fragment sequences obtained by 454 sequencing could be generalized to any transcription analysis.

### Sequence divergence

We studied the effect of sequence divergence by randomly mutating 3%, 6%, and 9% of the bases in a read following a Jukes-Cantor model of sequence evolution [21]. It should be noted that each read differed, thus our analysis does not reflect the effect of specific mutations in the genome. Rather we were interested in the general effect of mutations in the sequences. At the same way we evaluated effect of insertions introducing a single insertion in random position of each read, which size is 3%, 6% and 9% of the read length.

### Mapping of the reads

The in silico generated reads were mapped either to the transcriptome or the complete genome using different programs: BLAT [12] (parameters –oneOff 1), SSAHA2 [13] (parameters –skip 2, -diff 0, -kmer 13, -solexa for 35 bp and 50 bp reads; -454 for 100 bp reads), Bowtie [14] (parameters –k 1, -n 3, -e 2000), SeqMap [15] (number of mismatches = 3), MAQ [16] (parameters –n 3, -e 2000) and the commercial tool CLC NGS Cell [www.clcbio.com] (algorithm: *clc_ref_assembly_long*, parameters: -r ignore). To keep the results most comparable as possible we set for each program only the essential parameters and assigned 3 mismatches to the programs that needed this parameter (Bowtie, SeqMap, MAQ), which is also the maximum number of mismatches allowed by these three programs.

Then we mapped the reads first against the transcriptome and then against the genome of the species considered. For each program, we used the coordinates of the best unique hit to determine if each read is mapped to the correct gene.

The proportion of correctly mapped reads e_obs_ was determined for each gene and we produced, for each program, the percentage of genes that recover less than 50% of the expected expression (e_exp_ = 200).

### Exon analysis

This was done to confirm that Bowtie has difficulties mapping reads that span two exons. Rather than generating sequence reads from transcripts, we only used exons of genes with two exons. Hence, the reads did not contain exon junctions. We generated for each exon 100 reads of lengths 35, 50 and 100 bp. These reads were mapped with Bowtie against the transcriptome and the genome of *D. melanogaster*.

PERL scripts have been used for filtering the transcripts, generating the reads and counting the number of correctly mapped reads. They are available in the supplementary Material S1.

## Supporting Information

Table S1 (0.03 MB DOC)

Table S2 (0.03 MB DOC)

Table S3 (0.03 MB DOC)

Material S1 (0.01 MB ZIP)
