# Correction: Mapping Accuracy of Short Reads from Massively Parallel Sequencing and the Implications for Quantitative Expression Profiling

**DOI:** 10.1371/annotation/8d1d51b9-81be-4351-ba45-27e3afdea13a

**Published:** 2009-10-09

**Authors:** Nicola Palmieri, Christian Schlötterer

Table S3 is incomplete. Please view the complete file here: 
