# Supplementary material for: Mapping Accuracy of Short Reads from Massively Parallel Sequencing and the Implications for Quantitative Expression Profiling
Source: PLoS One. 2009 Jul 28;4(7):e6323. doi: 10.1371/journal.pone.0006323 (PMC2712089; doi:10.1371/journal.pone.0006323)
Supplement: Table S1 — (0.03 MB DOC) [file pone.0006323.s001.doc]

**Table S1** Number of transcripts used in the simulations.

| **Organism** | **Before filtering^1^** | | **After filtering**^2^ | |
| --- | --- | --- | --- | --- |
|  | **Number of transcripts** | **Total size (Mbp)** | **Number of transcripts** | **Total size (Mbp)** |
| *S. cerevisiae* | 6532 | 9.6 | 6129 | 9.3 |
| *D. melanogaster* | 20822 | 56.9 | 13653 | 33.4 |
| *A. thaliana* | 33122 | 54.0 | 26901 | 44.9 |
| *H. sapiens* | 56155 | 132.9 | 21062 | 60.9 |

^1^ includes overlapping transcripts and all isoforms ^2^ without overlapping transcripts and only the longest isoform
