# Supplementary material for: Mapping Accuracy of Short Reads from Massively Parallel Sequencing and the Implications for Quantitative Expression Profiling
Source: PLoS One. 2009 Jul 28;4(7):e6323. doi: 10.1371/journal.pone.0006323 (PMC2712089; doi:10.1371/journal.pone.0006323)
Supplement: Table S2 — (0.03 MB DOC) [file pone.0006323.s002.doc]

**Table S2** Percentage of mapped and correctly mapped exonic reads with BowTie. (For further information see Materials and Methods)

| **Database** | **Transcripts** | | | **Genome** | | |
| --- | --- | --- | --- | --- | --- | --- |
| Read length | 35 bp | 50 bp | 100 bp | 35 bp | 50 bp | 100 bp |
| Mapped (%) | 100.00 | 100.00 | 100.00 | 100.00 | 100.00 | 100.00 |
| Correct (%) | 98.61 | 98.70 | 98.88 | 98.05 | 98.24 | 98.53 |
