# Supplementary material for: Mapping Accuracy of Short Reads from Massively Parallel Sequencing and the Implications for Quantitative Expression Profiling
Source: PLoS One. 2009 Jul 28;4(7):e6323. doi: 10.1371/journal.pone.0006323 (PMC2712089; doi:10.1371/journal.pone.0006323)
Supplement: Table S3 — (0.03 MB DOC) [file pone.0006323.s003.doc]

**Table S3** Features of the different mapping software

| **Program** | **Features** |
| --- | --- |
| BLAT | Not good if the similarity between read and reference is less than 95%, allows spliced alignments |
| SSAHA2 | Allows spliced alignments |
| Bowtie | Allows a maximum of 3 mutations, random assignment of unambiguous reads, no gaps, no spliced alignments |
| SeqMap | Allows a maximum of 3 mutations, no gaps, no spliced alignments |
| MAQ | Allows a maximum of 3 mutations, random assignment of unambiguous reads, no gaps, no spliced alignments |
| CLC NGS Cell | Allows spliced alignments |
