# Supplementary material for: Correction: Mapping Accuracy of Short Reads from Massively Parallel Sequencing and the Implications for Quantitative Expression Profiling
Source: PLoS One. 2009 Oct 9;4(10):10.1371/annotation/8d1d51b9-81be-4351-ba45-27e3afdea13a. doi: 10.1371/annotation/8d1d51b9-81be-4351-ba45-27e3afdea13a (PMC2763894; doi:10.1371/annotation/8d1d51b9-81be-4351-ba45-27e3afdea13a)
Supplement: Supplementary file 1 [file pone.8d1d51b9-81be-4351-ba45-27e3afdea13a.s001.doc]

**Table S3** Features of the different mapping software

| **Program** | **Features** |
| --- | --- |
| BLAT | Not good if the similarity between read and reference is less than 95%, allows spliced alignments, report all possible alignments, gaps allowed |
| SSAHA2 | No maximum number of mismatches, report all possible alignments, gaps allowed |
| Bowtie | Allows a maximum of 3 mismatches in the seed (first 28 bp by default), random assignment of ambiguous reads by default but it can report all possible alignments, no gaps |
| SeqMap | Allows a maximum of 5 mismatches in the read, random assignment of ambiguous reads by default but it can report all possible alignments, gaps allowed |
| MAQ | Allows a maximum of 3 mismatches in the seed (first 24 bp), random assignment of ambiguous reads by default but it can report all possible alignments, no gaps |
| CLC NGS Cell | No maximum number of mismatches, random assignment of ambiguous reads by default but it can ignore them, gaps allowed |
